# Silencing of RIPK4 inhibits epithelial-mesenchymal transition by inactivating the Wnt/β-catenin signaling pathway in osteosarcoma

**DOI:** 10.3892/mmr.2022.12585

**Published:** 2022-01-04

**Authors:** Zhigang Yi, Yanchuan Pu, Ruoyan Gou, Yonggang Chen, Xiaojun Ren, Wenzhong Liu, Ping Dong

Mol Med Rep 21: 1154-1162, 2020; DOI: 10.3892/mmr.2020.10939

Subsequently to the publication of the above article, the authors have realized that the same western blotting data shown for the vimentin bands for the U2OS cell line in Fig. 3B on p. 1159 had inadvertently been re-used for the β-catenin bands for the U2OS cell line in Fig. 5A on p. 1161.

The authors have re-examined their original data, and have realized that Fig. 5 was assembled incorrectly. The corrected version of Fig. 5, showing the correct β-catenin bands for the U2OS cell line, is shown opposite. Note that this error did not quantitatively affect either the results or the overall conclusions reported in this paper. The authors are grateful to the Editor of *Molecular Medicine Reports* for allowing them this opportunity to publish a Corrigendum, and they apologize to the readership for any inconvenience caused.

## Figures and Tables

**Figure 5.**
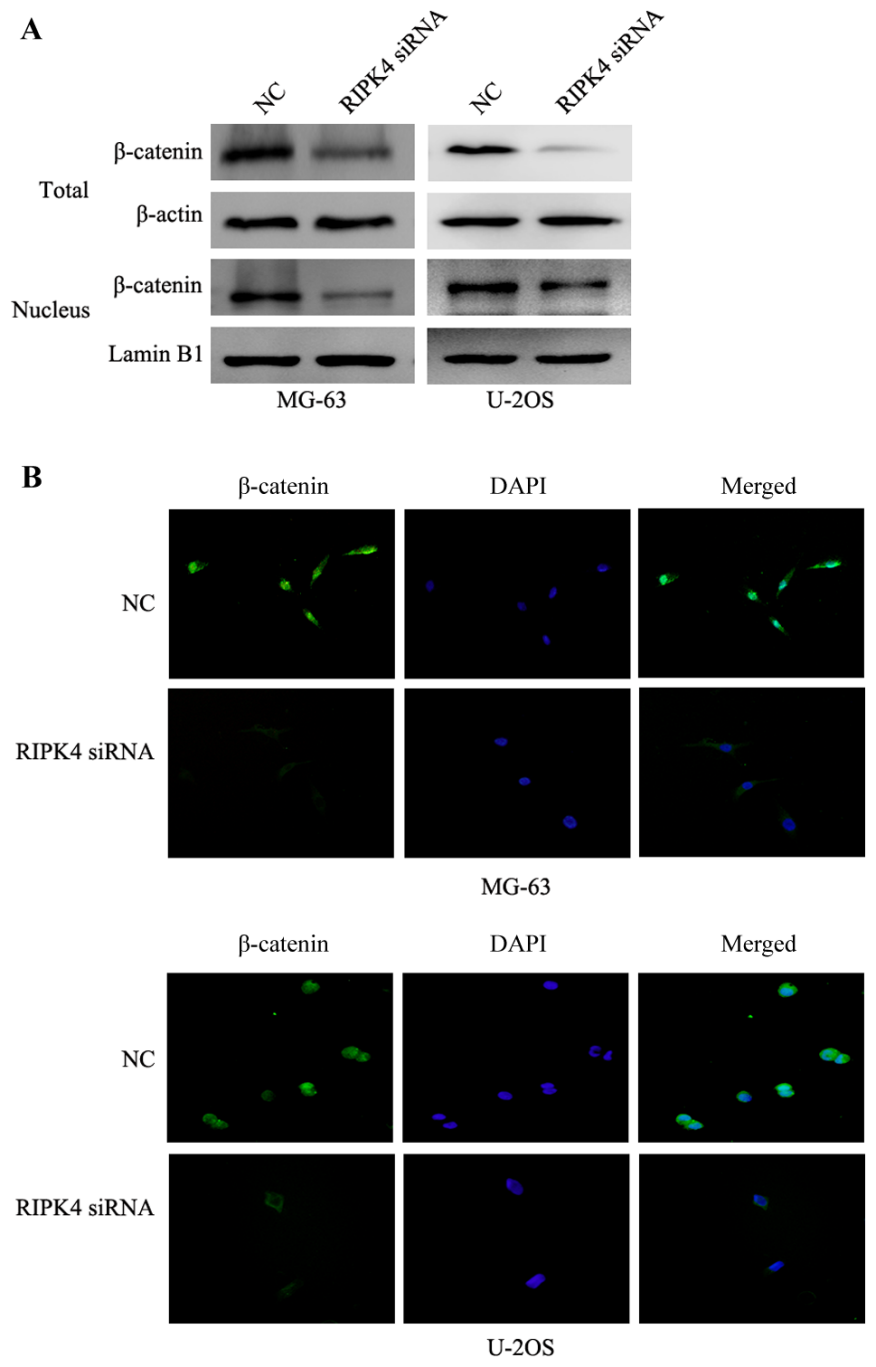
Silencing of RIPK4 suppresses the inactivation of the Wnt/β-catenin signaling pathway. (A) Western blot analysis revealed that RIPK4 silencing decreased total and nuclear β-catenin expression in MG-63 and U2OS cells. β-actin was used as the loading control for total expression. Lamin B1 was used as a loading control in the nucleus. (B) Immunofluorescent staining revealed that RIPK4 silencing reduced the levels of β-catenin. The targeted proteins were stained green and the nuclei were stained with DAPI (blue; magnification, ×200). RIPK4, receptor interacting protein kinase 4; DAPI, 4,6-diamidino-2-phenylindole; NC, negative control; siRNA, small interfering RNA.

